# Evaluation of Relation between Anthropometric Indices and Vitamin D Concentrations in Women with Polycystic Ovarian Syndrome

**Published:** 2014-09

**Authors:** Roya Faraji, Seyedeh Hajar Sharami, Ziba Zahiri, Maryam Asgharni, Ehsan Kazemnejad, Shirin Sadeghi

**Affiliations:** Reproductive Health Research Center, Guilan University of Medical Sciences, Rasht, Iran

**Keywords:** Poly Cystic Ovarian Syndrome, Androgen, Insulin Resistant, Vitamin D

## Abstract

**Objective:** To determine the association between serum 25 – hydroxy vitamin D concentration and anthropometric indices in patients with polycystic ovarian syndrome (PCOS).

**Materials and methods:** This is a descriptive cross – sectional study which was carried out on women with PCOS aged 19-39 years old referred to an infertility clinic of Alzahra Hospital, Rasht, Iran during September2011- March2012. The study was conducted based on the Rotterdam criteria. Exclusion criteria were hyperandrogeniema and thyroid dysfunction. The data were gathered through an interview with focus on demographic characteristics and history of infertility. The height, weight and waist and hip circumferences were measured and BMI had been calculated. Also, blood sample had been checked to indicate the level of hydroxy vitamin D. While all statistical analyses were carried out using software package used for statistical analysis (SPSS) version 16 (SPCC Inc., Chicago, IL, USA).

**Results:** Over 68% of patients had vitamin D deficiency (Vit D<15). Level of vitamin D had a significant correlation with waist circumference (p<0.02), height (p<0.001) and waist-to-hip ratio (WHR) (p<0.007).

**Conclusion:** Based on the anthropometric indices, it seems that we can predict the level of 25-hydroxyvitamin D in women with PCOS.

## Introduction

Recently, investigators have focused on vitamin D due to a significant deficiency of this vitamin not only in adults but also in pediatrics (1). 

Pervious studies found vitamin D deficiency in the pathogenesis of the metabolic syndrome (2), and a cohort study demonstrated association of obesity with decreased level of 25–hydroxy vitamin D in patients with polycystic ovarian syndrome (PCOS) (2-3). Also, another study (limited investigations what do you mean by this??) showed that vitamin D deficiency in patients with PCOS is associated with insulin resistance and diabetes type II (4, 5).

Wehr et al. reported 25-hydroxy vitamin D [25(OH) D] deficiency in 72.8% of PCOS women and pointed that PCOS women with metabolic disorders had less 25(OH) D concentration as compared with PCOS women without metabolic disorders. They also mentioned that 25-hydroxy vitamin D and body mass index (BMI) were independent predictors for PCOS, whereas there negative correlation between vitamin D with BMI, waist circumference, and waist-to-hip ratio (WHR) (6).

However, Hosseinpanah et al. revealed that metabolic disorders were not prevalent in PCOS group as compared to normal women (7). 

Furthermore, The results of studies conducted for school-age, reproductive age or menopause women suggested that vitamin D deficiency was highly prevalent (8- 12).

Although, there is no previous investigation on vitamin D deficiency in PCOS women living in north part of Iran, Maddah et al. showed that 84.7% of urban and 79.5% of rural menopause women suffered from vitamin D deficiency, indicating a life-threatening problem (10) . 

Therefore, the aim of this study was to determine the association between serum 25–hydroxy vitamin D concentration and anthropometric indices in patients with PCOS.

## Materials and methods

This is a descriptive cross sectional study conducted on women with PCOS aged 19-39 years old referred to an infertility clinic of Al-Zahra Hospital in city of Rasht, north part of Iran. This study was approved by the Ethics Committee of Gulian University during Agust 2011. All participates provided a signed informed consent.

The diagnosis of PCOS was based on the presence of 2 out of 3 features of Rotterdam criteria (2003). This criteria consists of different clinical manifestations of both oligo-ovulation or hyper androgenism (clinical or laboratory), including hirsutism, acne, hair loss, elevated testosterone/dehydroepiandrosterone (DHEAS), as well as morphologic view of PCOS in ovarian sonography. Exclusion criteria were presence of Cushing syndrome, congenital adrenal hyperplasia (CAH), hyperprolactinemia, impaired thyroid tests, virilizing tumors, diabetes mellitus, oral contraceptives (OCP) and progesterone consumption six month before starting this study, breast feeding period, history of bone fracture, renal or liver diseases six month before starting this study, as well as alcohol/steroid drugs consumption. Therefore, at the initiation, laboratory tests and clinical manifestations were carried out, and patients with impaired results were referred to an endocrinologist. 

Data consisting of demographic characteristics (age, educational level and occupation), anthropometric indices and history of infertility (lack of pregnancy for a year without contraceptive drugs consumption) were gathered through an interview conducted by a trained researcher. Weight was measured in light indoor clothing and barefoot or with stockings and height was measured in aerect position by tape meter. Then, using the following formula to calculate the body mass index: BMI= kg/m^2^, in which weight (kg) was divided by height (m) squared. Based on BMI findings, we divided the participants into two following groups: (i) less than 24.99kg/m^2^ as normal patients and (ii) more than 24.99kg/m^2^as obese ones.

In order to calculate waist-to-hip ratio (WHR), waist circumference measurement was obtained just above the iliac crest and exactly under navel by a tape meter, while hip circumference measurement was obtained when the tape meter was positioned horizontally around the maximum circumference of the buttocks. 

Serum concentration of 25(OH) D was measured by Kobas kit (Akbarie Company, Germanny) through (Eclia method). Vitamin D deficiency were indicated as <15ng/ml and sufficiency was defined as >30ng/ml of serum vitamin D Normalized distribution of quantitative variables was indicated by Kolmograv-Smirnov test and the Spearman correlation coefficient assessed vitamin D concentration in univariate analysis of quantitative variables. All statistical analyses were carried out using software package used for statistical analysis (SPSS) version 16 (SPCC Inc., Chicago, IL, USA). Also, independent T-test and ANOVA were carried out to compare mean vitamin D in categorical variables. 

Stepwise multiple linear regressions were performed to control effects of confounding variables in relation with vitamin D concentration. A p-value of 0.05 was considered statistically significant (two-tailed).

## Results

A total of 77 patients with PCOS were assessed based on anthropometric indices and serum 25-hydroxy vitamin D concentration. The mean age were 24.9±5.2 (age range 14.36), while 60% of patients were married and have diploma or lower educational level. Also, 65% were household or unemployed. Table 1 demonstrates demographic characteristics and anthropometric indices of patients.

Our result showed that 88% of women had vitamin D deficiency (vitamin D< 15), and only in 9.3%, more than 30ng/ml of vitamin D concentration were reported.

**Table 1:** Demographic characteristics and antropometric indices

Mean age	24.9± 5.2
Single	26
Married	51
Less than diploma	25
Diploma	24
associate degree	7
Bachler of science	16
Master of science	4
Unemployed	11
Employee	17
Household	38
University student	11
Weight (kg)	72.82± 14.7
Height (cm)	159.17± 6.19
Waist circumference (cm)	87.90± 11.36
Hip circumference (cm)	107.67± 10.31
Waist to hip ratio	0.81
BMI (less than 24.99)	54
BMI (more than 24.99)	23

Table 2 shows frequency distribution of body mass index and vitamin D levels in both groups (according to BMI groups), which indicates in more than 50% of samples, vitamin D concentrations were insufficient or deficient. 

Also, results demonstrated that there are no significant differences between vitamin D concentration and demographic characteristics, including age, marital status, and history of pregnancy, reproductive status, educational level and occupation.

Pearson correlation coefficient showed that there are significant differences between vitamin D concentration with waist circumference (p=0.02), height (p=0.001) and WHR (p=0.007), whereas there are no significant differences between vitamin D with BMI and hip circumference.

**Figure 1:**
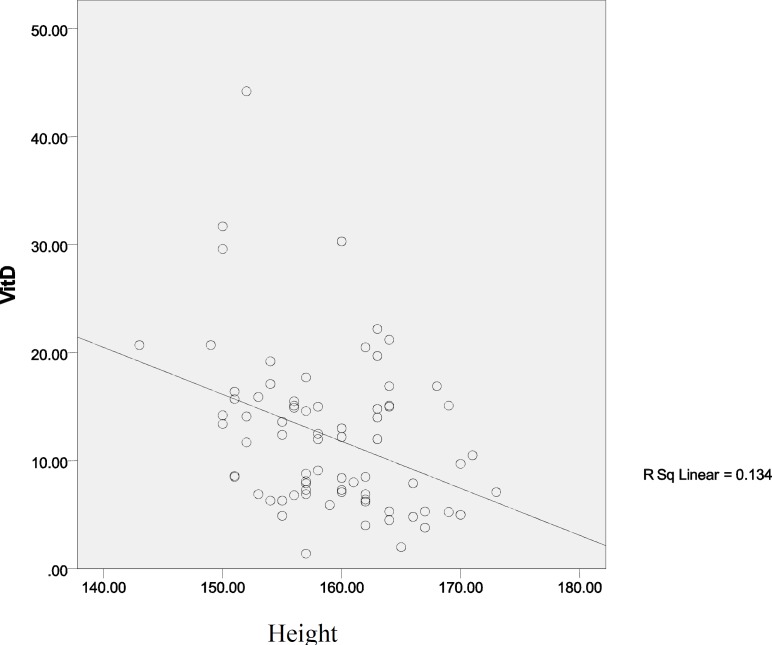
Linear relation between height and vitamin D concentration

**Figure 2:**
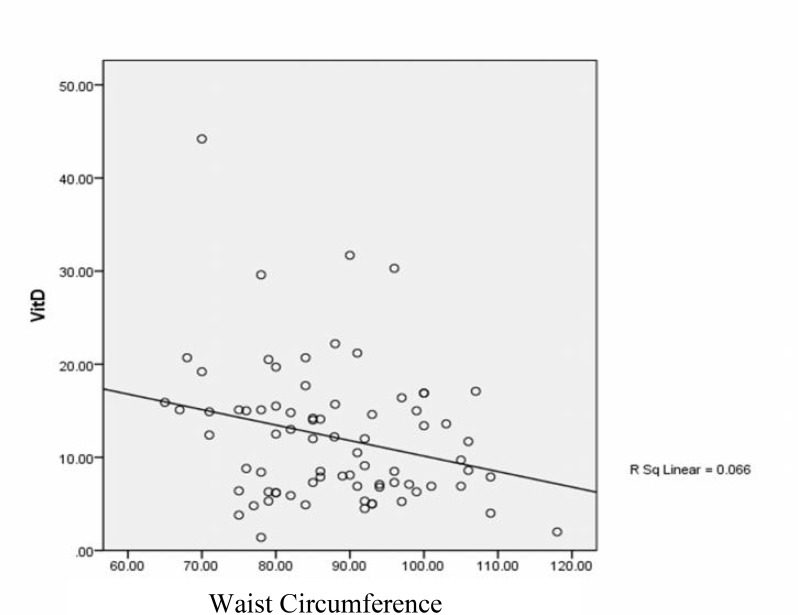
Linear relation between waist circumference and vitamin D concentration

**Figure 3:**
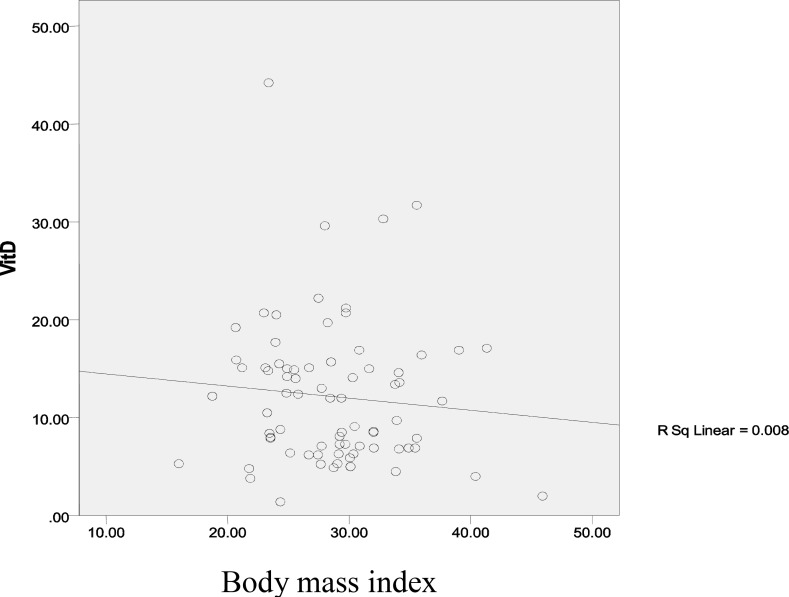
Linear relation between body mass index and vitamin D concentration

**Figure 4:**
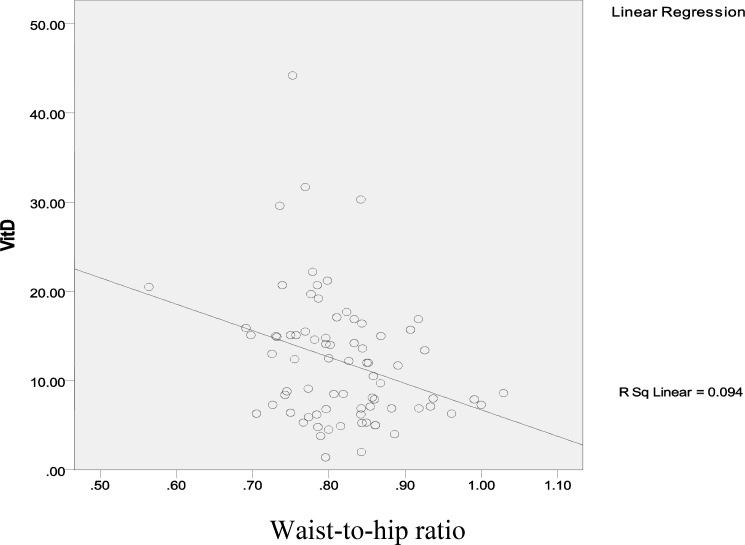
Linear relation between waist-to-hip ratio and vitamin D concentration

Figures 1-4 show linear relation between anthropometric indices and vitamin D concentration. 

**Table 2:** Frequency distribution of body mass index (BMI) and vitamin D levels among patients

		**Vitamin D levels**			
		**Deficient**	**Insufficient**	**Sufficient**	**Total**
BMI	<24.99 (%)	20 (29.4%)	2 (3.33%)	1 (33.3%)	23 (29.9%)
	>24.99 (%)	48 (70.6%)	4 (66.7%)	2 (66.7%)	54 (70.1%)
Total	n (%)	68 (100%)	6 (100%)	3 (100%)	77 (100%)

 To investigate the relation between anthropometric indices and vitamin D concentration in patients with PCOS, the effect of demographic characteristics was controlled, so stepwise multiple linear regression showed that height (p=0.0001) and WHR (p=0.008) were considered as predictive variables, in which one centimeter increase in height is associated with an -0.410 decrease in vitamin D concentration (CI 95%=0.165 and 0.655), while -27.15 decrease in vitamin D concentration would be resulted from 1 cm increase in WHR (CI 95%=-7.225 and -47.08).

## Discussion

It has been generally stated that more than 50% of PCOS patients are obese, and it seems that increased androgen and insulin resistance are the main etiology of the disease. According to previous results, vitamin D in obese women are less than normal level, and it should be noted that there are negative correlation between BMI and serum vitamin D in patients with PCOS (6, 13, 14).

Wehr et al. reported that there were significant differences between BMI in vitamin D deficient and vitamin D sufficient in women with PCOS. They showed that there was significant difference between 25-hydroxy vitamin D concentration and age, which is inconsistent with our results. Also, they mentioned that there were negative correlations between vitamin D concentration with waist circumference, WHR, systolic and diastolic blood pressure, as well as fasting blood sugar (FBS) (6).

In our study, there is no significant difference between BMI groups, whereas a significant difference expressed regarding to height between our study and Wehr’s study may be as a result of difference in sample size (6).

Furthermore, our results revealed that there are negative considerable differences between vitamin D concentration with height and WHR, which was similar with previous results indicating negative relations between vitamin D concentration with height, WHR, BMI and anthropometric indices (3, 6).

It should be mentioned that more than 0.85 waist-to-hip ratio could manifest lipid android distribution in body leading to hyperinsulinemia, glucose intolerance, diabetes mellitus and elevated androgen level (15). Although in similar studies inconsistent results were mentioned (16, 17). It seems that anthropometric indices could be influenced by other features besides vitamin D concentration and PCOS. 

Wehr et al. and Thomson et al. showed the prevalence of vitamin D deficiency in patients with PCOS as 67-85 % and 72.8%, respectively (6, 18), which was less than related values in our results. However, it should be noted that hirsutism in obese women with PCOS commonly occurred, which obliges them to avoid sun exposure and consequently leads to less vitamin D concentration. 

Mahmoudi et al. and Forouhi et al. expressed that there was strong relation between 25-hydroxy vitamin D concentration and BMI in patients with PCOS (3, 19); in addition, Wehr et al. reported that there was significant relation between vitamin D concentration with metabolic syndrome and insulin resistance in patients with PCOS (6). Kotsa et al. demonstrated that vitamin D supplementation had beneficial effect on insulin discharge and serum lipids in patients with PCOS (20); however, our results showed no significant difference in this regard.

Also, Vertsman et al. mentioned that obese women were less likely to show higher vitamin D concentration after exposure to sun light in comparison with normal weight women (21), while weight loss was the most efficient treatment in women with PCOS (6). 

Acorrding to previous investigations, there is no known accurate mechanism to define the relation between decreased level of vit D and insulin resistance. They mentioned that it could be as a result of effective influence of vitamin D on insulin function in which vitamin D could elevate the response of insulin receptor and consequently that insulin response could facilitate the enterenace of glucose to the cells. Also vitamin D canjutify the intra and outer cellular calcium which is mandatory for insulin process. Finally, as vitamin D could impact the immune system, vit D hypovitaminose could increasingly induce the inflammatory response which could be related to insulin response. In addition Wehr et al indicated that 25 hydroxy vit D deficiency could be associated with increased level of CRP (7, 13, 14, 22).

The limitations in our study were lack of assessment metabolic syndrome status, glucose and lipid indexes. Also, in our study, we didn’t include control group for comparing results. 

According to results, it seems that vitamin D deficiency, obesity and PCOS are related, in which vitamin D deficiency leads to obesity, insulin resistance and conseqently, PCOS. Therefore, we recommend further investigations regarding to vitamin D supplementation as a treatment for patients with PCOS and other influential factors such as calcium+ vitamin D, carbohydrate consumption and physical activity. 
